# Supplementary material for: Inverting the deficit model in global mental health: An examination of strengths and assets of community mental health care in Ghana, India, Occupied Palestinian territories, and South Africa
Source: PLOS Glob Public Health. 2024 Mar 4;4(3):e0002575. doi: 10.1371/journal.pgph.0002575 (PMC10911620; doi:10.1371/journal.pgph.0002575)
Supplement: S1 Text — Table B: Contributors Table. (DOCX) [file pgph.0002575.s001.docx]

## S1 TEXT – Inverting the deficit model in global mental health

**Table A** : Sources of Primary Data from analysis

| **Country and Title of study** | **Methodology used** | **Ethics status** | **Coauthors from this collaboration** | **Years** | **Published peer reviewed papers linked to this project** | **Funding** |
| --- | --- | --- | --- | --- | --- | --- |
| Ghana  *Mental Health and Poverty Project (MHaPP)* | Qualitative, semi-structured interviews | Approval by ethics committes of Kintampo Health Research Centre and University College London | Kenneth A Ae-Nigbise  Ursula M Read | 2007-2009 | Read UM Read, Adiibokah E, Nyame S. Local suffering and the global discourse of mental health and human rights: An ethnographic study of responses to mental illness in rural Ghana. Global Health [Internet]. 2009;5(1):13. Available from: https://doi.org/10.1186/1744-8603-5-13 | DFID |
| *Between Chains and Vagrancy: Living with Mental Illness in Kintampo, Ghana* | Ethnography: observation, interviews, focus groups |  | Ursula M Read | 2006-2009 | UM Read. No matter how the child is, she is hers”: Practical kinship in the care of mental illness in Kintampo, Ghana. Ghana Studies. 2013;15(103):33. | Economic and Social Research Council  PTA-031-  2005-00036 |
| India  *Exploring Community Mental Health Systems -A Participatory Health Needs and Assets Assessment in the Yamuna Valley, North India* | Participatory action research  Focus group discussion, Participatory rural appraisal and semi-structured interviews | Approval by Institutional Ethics Board of Emmanuel Hospital Association, New Delhi August 2019 (protocol 208) | Kaaren Mathias  Meenal Rawat  Sumeet Jain | 2018-2021 | Mathi Rawat M, Jadhav S, Bayetti C, Mathias K. A Qualitative Study to Explore Various Meanings of Mental Distress and Help-Seeking in the Yamuna Valley, North India. Indian J Soc Psychiatry [Internet]. 2021;37(4). | Mariwala Health Initiative |
| *Exclusion and inclusion of people with mental disorders in rural North India* | Qualitative – in-depth interviews | Approval by Institutional Ethics Board of Emmanuel Hospital Association, New Delhi April 2013 | Kaaren Mathias | 2014-6 | Mathias K, Jacob KS, Shukla A. “We sold the buffalo to pay for a brain scan”–a qualitative study of rural experiences with private mental healthcare providers in Uttar Pradesh, India. Indian J Med Ethics. 2019;4(4):282–7.  K. Mathias, K. Kermode, M. San Sebastian, M. Korschorke and I. Goicolea Under the banyan tree - exclusion and inclusion of people with mental distress in rural North India BMC Public Health 2015 Vol. 15 Issue 446 DOI: 10.1186/s12889-015-1778-2  Mathias, K., et al. (2018). "An asymmetric burden: Experiences of men and women as caregivers of people with psycho-social disabilities in rural North India." Transcultural Psychiatry 56(1): 76-102. | NA |
| Occupied Territories of Palestine  *Mental Health and Justice* | Semi-structured interviews  Participatory action research  Participant observation | Approval by King’s College London (HR-16/17-4222) and  Birzeit University (62610) | Weeam Hammoudeh  Suzan Mitwalli  Hanna Kienzler | 2017-2022 | Ki  Hammoudeh W, Kienzler H, Meagher K, Giacaman R. Social and political determinants of health in the occupied Palestine territory (oPt) during the COVID-19 pandemic: who is responsible? BMJ Glob Health. 2020;5(9):e003683.  Kienzler H. Mental health system reform in contexts of humanitarian emergencies: toward a theory of “practice-based evidence”. Culture, Medicine, and Psychiatry. 2019 Dec;43(4):636-62. | Wellcome Trust as part of the Mental Health and Justice project (203376/Z/16/Z) |
| South Africa  *Stakeholders' perceptions of child and adolescent mental health services in a South African district* | Interviews and focus groups | Biomedical Research Ethics Committee, Faculty of Health Sciences, University of KwaZulu-Natal (Reference number BE098/18). | Andre Janse van Rensburg | 2019 | Janse van Rensburg A, Kathree T, Breuer E, Selohilwe O, Mntambo N, Petrus R, Bhana A, Lund C, Fairall L, Petersen I. Fuzzy-set qualitative comparative analysis of implementation outcomes in an integrated mental healthcare trial in South Africa. Global Health Action. 2021 Jan 1;14(1):1940761 | PRogramme for Improving Mental health carE (PRIME). This work was financially supported by the UK Department for International Development (201446) |

**TABLE B:** Contributor table

| Initials | Affiliations | Member of global majority (person of colour) | At least 8 working or living in one of these four countries | Country of residence at paper inception/ Country team link | Contribution to this paper |
| --- | --- | --- | --- | --- | --- |
| KAA-N | Kintampo Health Research Centre | Y | Y | Ghana | Data collection and analysis; writing |
| LK | University of Ghana | Y | Y | Ghana | Data collection and analysis; writing |
| DT | Mindfreedom Ghana | Y | Y | Ghana | Data analysis |
| UMR | University of Warwick | N | Y | United Kingdom/ Ghana | Data collection and analysis; writing, Senior Author |
| PP | Burans, HCH | Y | Y | India | Data collection, analysis and writing |
| MR | Burans, HCH | Y | Y | India | Data collection, analysis and writing |
| SKS | Burans, HCH | Y | Y | India | Data collection |
| KJ | Centre for Mental Health Law and Policy, Indian Law Society, *Pune*, India | Y | Y | India | Data collection, analysis and writing |
| SS | Centre for Mental Health Law and Policy, Indian Law Society, *Pune*, India | Y | Y | India | Data collection |
| KM | University of Canterbury and Burans (HCH) | N | Y | New Zealand/ India | Data collection; analysis; writing; coordination |
| HK | King’s College London | N | Y | UK/ Occupied Palestine | Data collection and analysis; writing, Senior Author |
| SM | Birzeit University | Y | Y | Occupied Palestine | Data collection and analysis; writing |
| YR | Birzeit University | N | Y | Holland/ Occupied Palestine | Data collection and analysis |
| WH | Birzeit University | Y | Y | Occupied Palestine | Data collection and analysis; writing |
| AvR | University of KwaZulu-Natal | N | Y | South Africa | Data analysis; Writing |
| AK | Stellenbosch University, South Africa | Y | Y | South Africa | Writing |
| NB | University of Auckland | N | N | New Zealand | Data analysis and writing |
| SJ | University of Edinburgh | Y | N | Scotland | Analysis, Senior Author |
| DB | King's College London | N | Y | United Kingdom | Writing, Senior Author |
| RAB | University College London | Y | Y | United Kingdom | Writing, Senior Author |
