# Supplementary material for: Inverting the deficit model in global mental health: An examination of strengths and assets of community mental health care in Ghana, India, Occupied Palestinian territories, and South Africa
Source: PLOS Glob Public Health. 2024 Mar 4;4(3):e0002575. doi: 10.1371/journal.pgph.0002575 (PMC10911620; doi:10.1371/journal.pgph.0002575)
Supplement: Attachment — Submitted filename: 2 Response to reviewers T2T Community mental health systems.docx [file pgph.0002575.s002.docx]

**Response to reviewers T2T Community mental health systems**

**September 2023**

**Review Comments to the Author**

Please use the space provided to explain your answers to the questions above. You may also include additional comments for the author, including concerns about dual publication, research ethics, or publication ethics. (Please upload your review as an attachment if it exceeds 20,000 characters)

**Reviewer #1:** Dear Author, This paper is well written, however would like to get some clarification.

1. How does the team come to the conclusion of selecting 4 country case studies where 24 countries researchers are participating in the T2T programme.

*Response: We have clarified the selection of these countries in the text of Box 1 stating:*

*We chose to focus on the community health systems in Ghana, India, oPt and South Africa as four countries where participating team members had long-term engagements in and situated knowledge for at least eight years. While these four countries have diverse populations and geographies within and between them, they also share aspects of their political economies and colonial histories.*

2. How did the research team ensure that team members are not biased when writing interpretive case studies or sharing site information for case studies from each site.

*Response: This study has been written with the purposive inclusion of contributors with a strong contextual understanding of the four countries. Qualitative methodologies typically move away from the concepts of objectivity/ bias used in empirical research, to identify that all research is informed by the identity and positionality of a researcher and to reflexively considering how this influences data collection, analysis and interpretation (as we have done to some extent in the methods and in the newly added Contributor table).*

3. Is there a possibility to provide basic information on field or community researchers who collected primary data through in-depth interviews and Focus group discussion.

*Response: Thankyou for this suggestion. We have developed an additional Contributor table that provides an overview of the positionality of co-authors and other contributors to this study which we propose would be part of Supplementary materials for this publication. Please see the details in that table.*

4. Is there reason to leave out the influence of socio-cultural practice in informal care.

*Response - We recognise that care is embedded in social and political structures. We have added the following text to recognise this:*

**While communities are shaped by global socio-political forces, they also form local, contextualised responses to mental health challenges. Through cross-case analysis and discussions we identified the following assets that build community resilience and form a bedrock of mental healthcare in these countries. In keeping with our orientation to ‘informal’ assets beyond health and social care systems, we map these onto the primary social groupings within the study countries: the family and home; community and social support; faith-based care and traditional healers and non-profit organisations. The influence of historical, social, cultural, economic and political factors is threaded through all these domains shaping the possibilities for care and support in various ways.**

5. Suggestion:
 Write on limitations of these case studies

*Response - thank you for this suggestion. We have now included a new Methodological considerations section at the end of reported results which considers both strengths and limitations of case study methods.*

6. Improve the methodology by providing details on process in these collaborative case studies.

*Response - thank you for this suggestion. We have further edited the Methods section which provides details on the process of collecting and synthesising the case study data. It has now been further developed with the inclusion of the Contributor table in the Supplementary materials*

7. Describe how did these themes arrived from the data or discussion

*Response - thank you for this suggestion. The methods ‘box’ has been updated to refer to the analysis process used (reflexive thematic analysis), and this is now referenced.*

8. Discuss on political aspects in informal care (How local governance can help)

*Response - thank you for this suggestion. We have edited to include this text:*

**Our findings underscore that those designing programmes and policies need to engage with local historical and socio-political contexts as well as informal care practiced in communities, to ensure mental healthcare is relevant and acceptable (71,72). Design and delivery of mental health systems and care should be codesigned with communities and build on local assets (61,71).**

**Reviewer #2:** This paper starts from the position that the recent history of global mental health has tended to focus on the deficits in available formal health service provision, without acknowledging and valuing the important contribution of informal support that is provided to people with mental health needs in communities. It outlines the dominance of research and measurement of available support in the formal health/psychiatric sector; a point which is well justified, but there should perhaps be some recognition of the recent focus on social determinants of mental health, and the widespread adoption in global mental health research and in WHO and other UN guidance, of lived experience participation (though these principles are far from widely enacted in service provision in countries).

1) This has started to provide some counterbalance to the colonial histories that are well, if sometimes frustratingly briefly, described for such important issues. For example it is incomplete to leave the history of asylums in India with the East India company (even in a brief overview).

*Response: Thank you for this comment. We agree that there is an increased focus on social determinants and LE participation which warrants mention. We have edited the introduction and contexts of care sections to better represent the shift towards social health determinants and the collaboration of people with lived experience.*

*We have included this text in the opening paragraphs:*

**However, the framing and solving of the problems across global mental health has become more expansive in recent years, to recognise the central contribution of social determinants of health such as housing and social inclusion to poor mental health. This has been accompanied by the acknowledgement that when people with lived experience collaborate in the design of mental health care, services are more relevant, acceptable and equitable.**

*We have also updated the statements about the history of asylums in India to a more contemporary summary as pasted below:*

**In India, through the 19th and 20th centuries, government provided mental health care almost exclusively through a handful of large mental health care institutions which extended nationally, first through the East India company and later through the expansion of Western psychiatry and associated pharmacological treatments delivered through inpatient and outpatient care for people with severe mental health problems, although the total number of psychiatrists and in-patient beds has remained low (41) .**

2) While still very surprising, and true to the point being made, the statistic that 94% if the Indian mental health budget is allocated to just 2 institutions is not correct (line 194). This refers to the percentage of the direct budget only.

*Response - thank you. We have clarified this comment to read as follows:*

**In India, 94% of the 2022 direct mental health budget (i.e. allocated to the Ministry of Health and Family Welfare) was assigned to two tertiary care institutions, leaving 6% for implementation of district mental health programmes in the rest of the country (42).**

3) The use of the term 'care' sometimes grates when referring to abusive systems (lines 204/205 are examples).

*Response - thank you. We have reviewed the manuscript and changed this terminology to services in multiple instances, especially where it is clear that ‘care’ is not being provided.*

4) The arguments about the negative impact of structural adjustment, capitalism and neo-liberal economics provide a fair broad framework for understanding the context for some of the background drivers of mental health issues in countries, but the mechanisms of links between poverty, unemployment and economic inequity are not described in detail. Making these links explicit is important, otherwise the paper risks moving into polemic, especially as the range of examples of failure of the mental health system lacked a sense of direction. Is the key point the lack of investment, that the type of investment has been harmful, or that injustices of colonial history, neo-liberal capitalism or occupation are drivers of mental ill health, even as (not enough) international investment is made in service provision.

*Response - Thank you for this comment. We have extensively revised this section to try to highlight the role of neoliberal economics as both a distal health determinant and also as a contributor to poor quality health systems. Please see this whole section which has been updated.*

5) Some points are reasonable in themselves, but do not provide a rounded view of an issue, but rather are used to make a single isolated point. For example, the reduction of welfare benefits to being a system whereby people are viewed as 'one-dimensional income generating bodies' does not do justice to the evidence of benefits of social protection for people with enduring and disabling mental ill health - something that has been hard fought for.

*Response - thank you - we have edited this paragraph to address this point with the following text .*

6) There is a typo on line 273 making the sentence unclear.

*Response - thank you. This has been corrected.*

7) The paper is strongest in the section describing the influence of non-formal sectors. The case studies add enormously to this, and it would be important that they are easily accessible to the reader. The organising into families, communities, faith-based provides and NGOs feels logical, though it is not clear what the method of analysis was to reach this framework.

Response – Thank you. We have provided further detail on our approach to analysis in the methods. We have also added a sentence to explain how these groupings were delineated based on the primary social groupings within the study countries. We have also been more explicit in the introduction to our approach to ‘community mental health’ which in this paper we approach not as a formalised ‘system’ but as a patchwork of resources and supports drawing on Seye Abimbola’s call for attention to locally embedded ‘organic’ versus externally driven ‘surgical’ interventions.

8) Peer support is mentioned positively throughout these results, and given the current interest in this area in research, it was slightly surprising that this was not identified as a separate section to report on. A clearer explanation of how the frame for reporting results was arrived at would help the reader to understand questions like this.

*Response: Thank you for this. The core premise of this paper is that peer support is active and evident in informal ways in communities. The focus on ‘peer support’ as a formalised ‘intervention’ can overshadow these informal social networks which happen organically with limited outside intervention. We have edited this paragraph to clarify as follows:*

**Formalised ‘peer support’ in which people with lived experience of mental illness are brought together to provide mutual support and advice, is not widely available in many of the study settings. However, our research uncovered organic forms of mutual support among people with lived experience and caregivers. These could cohere within existing social settings such as caregivers meeting within a prayer camp in Ghana, patients meeting at a mental health clinic or young people living with mental illness encountering others through social media.**

9) The Country Profiles are very strong, and more direct comparisons of similarities and differences would have been a useful way to anchor the findings more concretely in the data.

Response: We have edited our methods and results to clarify how we anchored these findings. Here is one example from our text that does this:

**Key themes that emerged from our cross-case analysis were discussed in a series of recorded meetings. We found that the socio-political health determinants of global economic structures and colonisation in all four countries exert significant influence on local community health systems. Comparing and contrasting findings across the case studies, we found that key assets for community mental health include care from family and community members, and support from non-profit organisations and religious and faith-based organisations.**

10) While it is not the primary purpose of this paper to assess quality of care in different settings formally, given the premise of the paper (that community settings provide care that forms an under-appreciated asset), some comment on the degree of human rights protection or abuse in different settings (including access to the right to health) would have been useful. This does arise in the discussion where oversight of interventions is suggested by the informal sector through localised accountability mechanisms.

*Response - Thank you for this comment. We have added a paragraph to discuss how the informality of these supports may mean that accountability and oversight is limited and also suggest where and how some localised accountability mechanisms operate.*

11) In the discussion, the point that any development of projects (or research work) should properly understand the community assets in the context of the new provision is well made, and directly follows on from the results. The assertion of the importance of co-production in the discussion is important, though not so clearly coming out of the analysis results. Perhaps the logic of this link could be made more strongly.

*Response - Thanks for this comment. We have reviewed our findings and added the following to the results to underline how families often coproduce care with affected family members. In our discussion we have also noted the following:*

**Participatory approaches that co-produce policies or services with people with mental health conditions are not well represented in any of these four community mental health systems or in the knowledge described above. Co-production involves those traditionally with lived experience of mental distress in designing, implementing, delivering and evaluating relevant care, resources and services and ensure that services are person-centred, cost-efficient, innovative and equitable.**

12) The discussion is strong when it attempts to link the use of a community asset approach with engaging with formal services, recognising that these formal and informal assets are not opposed to each other or mutually exclusive. Evidence shows that people make active choices about where they seek support, and often manage to combine accessing care from different sectors. Reference to the recent Gureje et al trial of primary care and traditional healer collaboration would have made sense.

*Response – thank you for this comment. We have revised the discussion substantially to now acknowledge the interaction and integration of formal and informal care that happens in communities. We have added reference to the Gureje et al trial in this section.*

13) The conclusion is clear and makes the case for inverting the current model of GMH well, though recognising and celebrating the substantial gains already made in the field (largely driven but critical literature and representative organisations of service users) would also be fair and aligns to the discussion. The principle of understanding and mobilising local assets routinely, while avoiding losing their nuance is an important one, and the assertion in the conclusion that social and structural drivers of mental distress need to be challenged while community assets are strengthened is compelling. These conclusions form a valuable contribution to these live debates.

*Response- Thank you.*

**Reviewer #3:** This is an important paper that a) examines the strengths/assets of “informal” community mental health care systems (shaped by sociopolitical context); and b) propose an alternative to conventional GMH interventions: to invert a “deficit model”. The authors rely on findings (experiential knowledge) from Ghana, India, oPt, and South Africa. Core to their argument is the idea that to strength community assets (care from family & community, NGOs, and religious organisations) will support reciprocal relationships between formal and informal sectors, and thus, the co-production of knowledge and practice. Apart from the relevance of the content, the fact that the paper is written as a community of practice is a valuable example of how to challenge some hegemonic academic conventions. I think that starting with the historical and “global” sociopolitical shapers of local community life is a strength of the paper (placing communities as both victims of such violent processes, as well as key agents of resistance to them).

I am a critical scholar based in a geographical region that is different than those examined in the paper. From such positionality, I can offer some feedback on a manuscript that already promises to make a much-needed contribution.

1) The paper focuses in four specific countries and in discussions specific to “global mental health”, citing literature constrained to such scope. This makes perfect sense; yet, the authors may want to consider knowledge from other regions (e.g. Latin America, although a Colombian experience is in fact included in the discussion). Especially considering how critical Latin American traditions regarding “community” seem to have have shaped arguments made in some of the cited references; and – I intuit- is epistemologically close to the selected method for producing knowledge in this study. For example, calls to invert “deficit” models have been made many decades ago (e.g. Fals-Borda in sociology; Freire in critical education; Quijano in relation to coloniality; Martín-Baró and Montero, in liberation and community psychology; Breilh in critical epidemiology). Of course, very important African and Asian thinkers have made relatively similar arguments, focused on their respective contexts. Overall, diverse authors within community and critical psychology tend to stress the idea of moving beyond deficit models (e.g. Kagat et al., 2019; Berroeta, 2014; Capella & Jadhav, 2020; Greeson et al., 2014, many others). I am not suggesting to cite this work, but to highlight that claims against “deficit” models have been around for a while (and that they have been conveniently ignored by groups with epistemic power, which makes the authors´ argument relevant and necessary when discussing GMH in present times).

*Response - Thank you for this comment. We are aware of and acknowledge the relevance of critical psychological and Latin American literature on the rejection of the deficit model, and the importance of re-centering embodied knowledges. We also acknowledge the critical political act of explicitly citing these scholars within work like this, and thank you for giving us the opportunity to do so. We have included our response which makes reference to two schools of thought on this idea: knowledges born in struggle (Martin-Baro and Biko) and Embodied local knowledge (Freire). Please see page 5 for the new text.*

2) It may be beyond the main focus of the paper, but claims of themes “emerging” from the data (e.g. p3, p.9) have been contested on epistemological grounds (see Braun & Clarke, 2021).

*Response: Thank you. We recognise this critique and share Braun and Clarke’s scepticism - we have amended this to describe generating themes from the data.*

3) Authors already mention this partially, yet some readers would appreciate a sentence or two explaining more operatively (i.e. at the level of procedure) what exactly is the “sociopolitical wheel” and how was it utilized in the study (p.9).

*Response: Thank you. We have added to the box which describes the methods, we have expanded on this, citing the work of Nancy Kreiger and others who draw our attention to the importance of considering the determinants, of social determinants – which is the foundation of the socio-political wheel. We have also provided the wheel as Figure One to be placed in the paper.*

4) Authors explain well how they produced knowledge; yet, one sentence felt a bit ambiguous: “we drew on our local networks of practitioners and researchers to analyse the four country reports….”(p.9). Which people made up such local networks in each country, specifically?

*Response: Thank you. We have now added a Contributor table in Supplementary materials which addresses this gap.*

5) The paper does not seem to include any primary data (i.e. participants´ quotes) from Ghana? (I apologize if it does and I missed it).

*Response: Thank you for this comment. Primary data is indeed presented from Ghana however this is mainly in the form of ethnographic description rather than interview quotes. Although we also have a wealth of interview data we would contest a hierarchical approach where interview data is considered methodologically superior to ethnographic observation since interviews are recognised as performative dialogue within limited parameters. The ethnographic method on the other hand is particularly suited to observing ‘taken for granted’ practices in context which might not be made explicit within interviews.*

6) Again, this point of discussion may go beyond the scope of the paper; yet, the authors discuss colonialism, capitalism and neoliberalism as “determinants” of MH; from the perspective of Collective Health (Breilh, 1977, 2021) – and its articulation into Collective “Mental” Health (Capella, 2023 – in Spanish), these would be part of dialectical processes of “social determination”, not “determinants”.

*Response: We appreciate this insight and agree that the linear framing of ‘determinants’ does not reflect the dynamic dialectical and relational operation of social and historical forces, as well as the foundational aspects of the model used to shape data analysis. We have edited our text to reflect this, including mention of Yates-Doerr’s critique.*

8) The positive role of NGOs is an important part of findings, as they “carry a substantial service burden in the absence of government systems” (p.22). Authors adequately addressed capitalism and neoliberalism in previous sections; yet, they may want to consider a more explicit link between these and the more problematic aspects of NGOs; e.g. as a well-intended “patch” solution to the inherent contradictions of capitalism (and to specific governments who fail to guarantee basic rights); as potential sources of neocolonial cultural globalization; agents of alienation and ideologisation, etc. (of course, none of this denies their monumental work in helping people where the state fails to do so).

*Response - Thank you for the insightful comment. We have included a paragraph in the discussion on p24 to highlight the risk that NGOs spread neoliberal ideology and perpetuate colonialism in LMICs. We use the point to further emphasize the need to change global funding structures to move away from HIC-led and funded projects and towards locally co-produced initiatives that focus on strengthening community assets. We have also moved the paragraph on the economic drivers of mental healthcare to follow so that the discussion moves from discussing the socio-political economy to focusing on community assets. The added text is pasted below:*

*Similarly, while we have identified NGOs as an asset to fill the gap left by insufficient formal services, they also risk importing the interests, ideologies and historical colonial power structures of their high-income funders, perpetuating colonial and neoliberal mental health systems. With 85% of all global health institution headquarters located in HICs, and 83% of global health leaders from high income countries, there can be a disconnect between NGO decision-makers and local priorities. To truly support the coproduction of mental healthcare and strengthening of local assets there needs to be a shift in global mental health financing so that resources and power are redistributed to ensure mental healthcare systems are run and lead by the communities they serve.*

I enjoyed reading this manuscript, and I thank the authors for the chance to learn from their valuable work.
