# Supplementary material for: Inverting the deficit model in global mental health: An examination of strengths and assets of community mental health care in Ghana, India, Occupied Palestinian territories, and South Africa
Source: PLOS Glob Public Health. 2024 Mar 4;4(3):e0002575. doi: 10.1371/journal.pgph.0002575 (PMC10911620; doi:10.1371/journal.pgph.0002575)
Supplement: Attachment — Submitted filename: Responses to reviewers PLOS GPH final with refs.docx [file pgph.0002575.s003.docx]

Responses to reviewers

Dear Editor,

Thank you for the further thoughtful suggestions from reviewers. Please find below our responses to reviewer comments.

Yours sincerely

Rochelle Burgess (corresponding author)

Line 112: Add "and" before "make colonial assumptions" to complete the thought.

Response: we have made this edit.

Lines 152-154: I would reconsider what "invisible" means here. Following from historical work tracing the colonial drive towards community mental health in Africa (Quarshie, N. O. (2022). Psychiatry on a Shoestring: West Africa and the Global Movements of Deinstitutionalization. Bulletin of the History of Medicine, and other critiques of the neoliberal and fiscal considerations underlying deinstitutionalisation in the US and other countries, one could argue that the lack of visibility and acknowledgement of informal support is the paradoxical result of the importance of that support (1). The more a mental health system can rely on spontaneous support, the less inclined to measure and calculate this support. Ideally, that support can be invoked in moral terms, as a set of reciprocal obligations that characterise “community”. This is compounded by the lack of clear criteria to estimate when "mental health provision" is enough

Response: Many thanks for this point and pointing us to Quarshie’s important work.. While it raises interesting points about the promotion of family-care prior to the global mental health, it overlooks both the agency of actors in the global south in promoting community-based mental health care and the potential for community care to move beyond medicalised interventions to social inclusion and the promotion of the rights of people with mental health conditions. Quarshie’s argument does not allow for the potential for community led-care to be a site of transformation, as it is based on a rather incomplete logic of care that overlooks the ways in which community-led care can draw attention to a much fuller range of social, political and cultural concerns than simply access to medical treatment. Furthermore, we argue that = care rooted in cultural and social contexts and relationships when given sufficient resources and support has potential benefits which extend beyond the economic and medicalised logic of deinstitutionalisation and ‘access to treatment’. While engagement with these topics in full is beyond the scope of this paper, we have made reference to this work and re-articulated another importance of what community logics of care ultimately draw us to – not JUST care in the community, but a problematising of what counts as care in the first place. Our updated text as pasted below:

*While some have argued that transitions to community-based care, particularly in the context of the treatment of people with serious mental illness in the global south, is informed by a colonial logic designed to reduce dependence on state investment, this overlooks the ways in which community-led care could extend beyond economic and medicalised rationales focused on increasing access to biomedical treatment to a more expansive logic of care that includes addressing social, political and structural sources of violence and exclusion* (1,2)*. Ultimately, community led-care could lead not simply towards the delivery of community-based treatment, but a rearticulation of what counts as care in the first place.*

Line 58: Please clarify the concept of “community mental health systems”. What makes the dynamic support provided by communities a “system”? Is it its integration with a formal network of services? Is it its holistic or integrated nature? This is a unique conceptual proposition of the paper and requires more clarification.

Response: Thank you, defining community mental health systems is something we needed to include. We have updated this section with this text:

*“Community health systems are defined by Schneider and Lehmann as “the set of local actors, relationships, and processes engaged in producing, advocating for, and supporting health in communities and households outside of, but existing in relationship to, formal health structures”*(3)*. A community mental health system in this study includes both vertical (hierarchical) and horizontal (relational) elements that are both formal and informal and based on networking, acceptability, trust and reciprocity. It crosses across the boundaries of public agencies, levels of government, and/or the public, private, non-profit and civic spheres and is by definition “context specific and are influenced by local histories, economic and political systems, and social–cultural norms”* (3)*.*

Line 215: Please explain what “textured summaries” are and what makes them different from a “summary”. If this concept has already been operationalised in the literature, please add a reference.

Response: We have changed this text to read more simply saying:

Summaries of community mental health systems in each country were developed using primary data already collected by group members, most of whom had worked together before.

Line 240: Please explain “reflexive thematic analysis”.

Response: Reflexive thematic analysis follows a updated approach to thematic analysis by Braun and Clarke (2022). We have clarified this using the following text in the Methods box:

*using reflexive thematic analysis which pays attention to the subjectivity and positionality of the researcher and the development of themes grounded in shared meanings and concepts and analyzed through theoretical frameworks to identify and describe the existing informal and formal mental health care services* (4,5)*.*

Lines 345 – 349 (and others). I wonder if the authors are interested in updating their description of the health system in the oPt, more generally, in response to the current crisis. This is complex due to the ongoing nature of the conflict, so I defer the decision to the authors.

Response: We have updated this section with text under the headings of Colonialism and Neoliberal economics as pasted below

*In Palestine, decades of illegal military Israeli occupation, blockade, and Apartheid have led to economic devastation, resulting to eroded health and social protection systems* (6)*. In the Gaza Strip, the current siege and bombardment have brought its health system to its knees leaving thousands without access to any form of even basic medical care or support* (7)*. With a lack of medication, electricity, and water supplies, doctors are hardly able to provide even the bare minimum of healthcare to the population. In both the West Bank and Gaza Strip there are few resident mental health professionals making mental health services less accessible.*

Lines 592 – 606: There are two potentially contradictory arguments in this paragraph. One states that NGOs introduce fragmentation because they are not coordinated with governments. The premise is that a coordinated and unified care system is preferable over a fragmented one. But then there is another argument stating that the integration of NGOs into healthcare systems compromises their independence and voice in the representation of civil society. The premise is that NGOs are valuable because of their independence. There is a tension between these two arguments that should be developed or made explicit.

Response: Thank you to the reviewers for this important comment. We have acknowledged this tension which is hard to resolve in the context of global inequities – we have pointed to the need for funding to improve equity of access to the kind of interventions NGOs can provide but recognise that this may in turn influence the independence of such actors. We also differentiate between larger NGOs and smaller organisations or individuals and highlight the need to acknowledge and account for the contribution of the latter – this argument is informed by Abimbola’s writings on ‘surgical’ and ‘organic’ interventions (8).

I also suggest a thorough round of proofreading.

We have made substantial edits to remove some repetition and clarify the arguments.

*BIBLIOGRAPHY*

1.	Quarshie NO. Psychiatry on a shoestring: West Africa and the global movements of deinstitutionalization. Bull Hist Med. 2022;96(2):237–65.

2.	Burgess RA. Working in the wake: transformative global health in an imperfect world. Vol. 7, BMJ Global Health. BMJ Specialist Journals; 2022. p. e010520.

3.	Schneider H, Lehmann U. From community health workers to community health systems: Time to widen the horizon? Health Syst Reform. 2016;2(2):112–8.

4.	Braun V, Clarke V. Reflecting on reflexive thematic analysis. Qual Res Sport Exerc Health. 2019;11(4):589–97.

5.	Braun V, Clarke V. Thematic Analysis: A Practical Guide. SAGE Publications Ltd; 2021.

6.	Kienzler H, Amro Z. ‘Unknowing’and mental health system reform in Palestine. Med Anthropol Theory. 2015;2(3):113.

7.	World Health Organisation. https://www.emro.who.int/media/news/attacks-on-health-care-in-gaza-strip-unacceptable-says-who.html. 2023. Attacks on health care in Gaza Strip unacceptable, says WHO.

8.	Abimbola S. The uses of knowledge in global health. Vol. 6, BMJ Global Health. BMJ Specialist Journals; 2021. p. e005802.
